# Enhancement of Partial Volume Correction in MR-Guided PET Image Reconstruction by Using MRI Voxel Sizes

**DOI:** 10.1109/TRPMS.2018.2881248

**Published:** 2018-11-15

**Authors:** Martin A. Belzunce, Abolfazl Mehranian, Andrew J. Reader

**Affiliations:** School of Biomedical Engineering and Imaging SciencesKing’s College London – St. Thomas’ HospitalLondonSE1 7EHU.K.

**Keywords:** Image reconstruction, MR-guided reconstruction, partial volume correction (PVC), positron emission tomography (PET), voxel sizes

## Abstract

Positron emission tomography (PET) suffers from poor spatial resolution which results in quantitative bias when evaluating the radiotracer uptake in small anatomical regions, such as the striatum in the brain which is of importance in this paper of neurodegenerative diseases. These partial volume effects need to be compensated for by employing partial volume correction (PVC) methods in order to achieve quantitatively accurate images. Two important PVC methods applied during the reconstruction are resolution modeling, which suffers from Gibbs artifacts, and penalized likelihood using anatomical priors. The introduction of clinical simultaneous PET-MR scanners has attracted new attention for the latter methods and brought new opportunities to use MRI information to assist PET image reconstruction in order to improve image quality. In this context, MR images are usually down-sampled to the PET resolution before being used in MR-guided PET reconstruction. However, the reconstruction of PET images using the MRI voxel size could achieve a better utilization of the high resolution anatomical information and improve the PVC obtained with these methods. In this paper, we evaluate the importance of the use of MRI voxel sizes when reconstructing PET images with MR-guided maximum *a posteriori* (MAP) methods, specifically the modified Bowsher method. We also propose a method to avoid the artifacts that arise when PET reconstructions are performed in a higher resolution matrix than the standard for a given scanner. The MR-guided MAP reconstructions were implemented with a modified Lange prior that included anatomical information with the Bowsher method. The methods were evaluated with and without resolution modeling for simulated and real brain data. We show that the use of the MRI voxel sizes when reconstructing PET images with MR-guided MAP enhances PVC by improving the contrast and reducing the bias in six different regions of the brain using regional metrics for a single simulated data set and ensemble metrics for ten noise realizations. Similar results were obtained for real data, where a good enhancement of the contrast was achieved. The combination of MR-guided MAP reconstruction with point-spread function modeling and MRI voxel sizes proved to be an attractive method to achieve considerable enhancement of PVC, while reducing and controlling the noise level and Gibbs artifacts.

## I. Introduction

Positron emission tomography (PET) provides quantitative functional images. However, it is well known that PET suffers from poor spatial resolution, around 4 mm in clinical scanners, which results in quantitative bias when evaluating the radiotracer uptake in small anatomical regions. These effects due to the low resolution of the scanner are usually referred to as partial volume effects (PVEs) and can be defined as the apparent loss in intensity or activity of an object with positive contrast in the image, when it occupies only partially the sensitive volume of the imaging system [1], [2], which in PET is the tube of response (TOR) measured by two detector crystals. PVE occurs when two adjacent regions spill-over counts between them due to the low resolution of the scanner, therefore hot regions suffer a loss of intensity while cold regions show an increase in their intensities.

The main consequence of PVE is the introduction of a bias when the activity concentration in a specific region needs to be quantified. For example, in brain imaging the uptake in cortical gray matter is of interest and it has only a few mm width (from 1 to 4.5 mm) and, as a result, the quantification on this region is greatly affected by PVE [3], [4]. This effect is also important in other smaller regions of the brain, such as the striatum, which is of importance in the assessment of a number of neurological diseases, such as Parkinson’s and Alzheimer’s disease (AD) [5]–[8]. For this reason, it is important to correct for this effect with partial volume correction (PVC) methods.

The goal of PVC is to compensate for the effect of limited resolution in a PET scanner, restoring the true activity distribution quantitatively and qualitatively in the reconstructed image. These techniques can be applied on the reconstructed images or during the reconstruction process. The two main methods in the latter group are resolution modeling and penalized likelihood (PL) using anatomical priors. Resolution modeling is applied in statistical iterative reconstruction methods [9], where the spatial resolution of the scanner, characterized with the point-spread function (PSF), is incorporated in the system matrix to enhance the spatial resolution of the reconstructed images [10]–[12]. However, the enhancement of the contrast and improvement in the resolution of the reconstructed images comes at the cost of the introduction of Gibbs artifacts due to the irrevocable loss of high frequency components during the acquisition [12], [13]. Gibbs artifacts can lead to significant quantitative errors in small hot regions [14], such as tumors or gray matter structures in the brain, hence it is not in widespread use in clinical applications, especially in cases where good quantification is required rather than lesion detection.

The second type of PVC methods applied during reconstruction incorporate anatomical information to reduce the noise in the image and, at the same time, enhance boundaries between anatomical regions, under the assumption that there is a match between the boundaries in the molecular image and the anatomical image. These methods are mainly based on PL or maximum *a posteriori* (MAP) algorithms and a wide variety of methods have been proposed to incorporate anatomical information in the prior energy function [15]–[24]. The introduction of clinical simultaneous PET-MR scanners has attracted new attention to these methods and brought new opportunities to use MRI information to assist PET image reconstruction for improving PET image quality. In this context, MR images are usually down-sampled to the PET resolution before being used in MR-guided PET reconstruction [18], [23], [24]. However, the reconstruction of PET images at the MRI voxel sizes could achieve a better utilization of the high resolution anatomical information and improve the PVC obtained with these methods.

In this paper, we evaluate the importance of the use of MRI voxel sizes when reconstructing PET images with MR-guided MAP methods, specifically the modified Bowsher method [17]. However, when the PET reconstruction needs to be done in a higher resolution matrix than the standard, limited by the sampling of lines of response (LOR), a number of artifacts arise in the image reconstruction depending on the projector and system matrix used. We propose a method to overcome these difficulties and we employ it to perform MR-guided MAP reconstructions using the MRI image as an anatomical prior in its original resolution, with the aim to enhance PVC. The MR-guided MAP reconstructions were implemented with a modified Lange prior that included anatomical information with the Bowsher method. The methods were evaluated for reconstructions with and without resolution modeling for simulated and real brain data. The images were assessed quantitatively computing image quality metrics using six different brain regions of interest (ROI).

## II. Image Reconstruction in High Resolution Matrix

The Biograph mMR PET-MR scanner (Siemens Healthcare, Erlangen, Germany) was used to evaluate the problems that arise when reconstructing images in a higher resolution than the standard voxel size with the goal of using anatomical information, such as an MRI image, in its original resolution. The mMR scanner sinograms have a radial bin size of 2.045 mm and the standard reconstructed images have a $2.09\times 2.09\times 2.03$ mm^3^ voxel size.

**Fig. 1.**
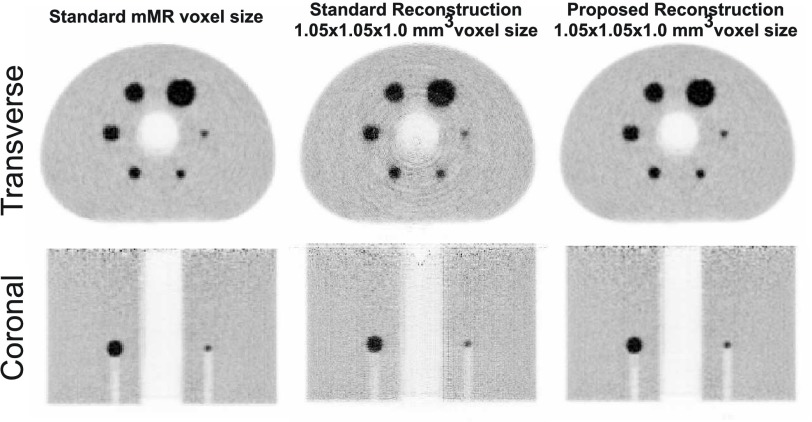
NEMA phantom scan reconstructed in the standard mMR voxel size and in a higher resolution matrix size ($1.0\times 1.0\times 1.0$ mm^3^ voxel size) with the standard MLEM reconstruction using a Siddon projector (middle) and the proposed method (right).

Fig. 1 shows a reconstructed image with the MLEM algorithm using the Siddon projector [25] for the standard and also a $1\times 1\times 1$ mm^3^ voxel size, where the reconstruction in a higher resolution matrix (middle column) introduces artifacts and gaps in the images. The main issue in this reconstruction is produced by the under-sampling of the projection data when using a Siddon projector with radial distances (separation between LORs) larger than the voxel sizes.

This issue can be solved by modifying the ray-tracing projector to take every pixel into consideration. For example, TOR [26] or multiray projectors [27] could be used. However, in these methods the complexity and the computational requirements are much higher than for a ray-tracing projector. In addition, in most of the cases the computational cost scales with the upsampling factor needed. For instance, in a multiray projector the number of rays needed depends on the pixel size of the reconstructed images making it not very efficient.

In this paper, we propose a simple, flexible, and efficient approach to overcome this issues, where an interpolation matrix is applied in image space before projecting the data with the Siddon algorithm. Therefore, this modified system matrix has two components

\begin{equation*} P_{\mathrm{ hr}} = X_{\mathrm{ lr}}D_{\rm hr\rightarrow {\mathrm{ lr}}}\tag{1}\end{equation*}

where $P_{\mathrm{ hr}}$ is the projection system matrix that projects a high resolution image into the standard sinogram size of the scanner, $X_{\mathrm{ lr}}$ is the Siddon projector for the standard voxel size and $D_{\rm hr\rightarrow {\mathrm{ lr}}}$ is a matrix that interpolates a high resolution image into the standard image size.

In the implementation of this method, special attention needs to be taken in the upsampling interpolation needed in the transpose of the projection matrix

\begin{equation*} P^{\mathrm{ T}}_{\mathrm{ hr}} = D_{\rm hr\rightarrow {\mathrm{ lr}}}^{\mathrm{ T}}X^{\mathrm{ T}}_{\mathrm{ lr}}\tag{2}\end{equation*}

where the upsampling matrix needs to be $D_{\rm hr\rightarrow {\mathrm{ lr}}}^{\mathrm{ T}}$, which is the transpose of the downsampling matrix $D_{\rm hr\rightarrow {\mathrm{ lr}}}$, to avoid having an unmatched projector/backprojector. A trilinear interpolation was used in our implementation of this method because it is a computationally efficient interpolation, even available in hardware on some platforms; and also, thanks to its simplicity, the upsampling matrix can be readily implemented as the transpose of the downsampling matrix. The latter can be achieved by computing the downsampling and upsampling weights starting from the high resolution voxel coordinates. This way, each voxel of the high resolution image is related to the same eight nearest neighbors of the low resolution matrix in both the upsampling and downsampling operations.

**Fig. 2.**
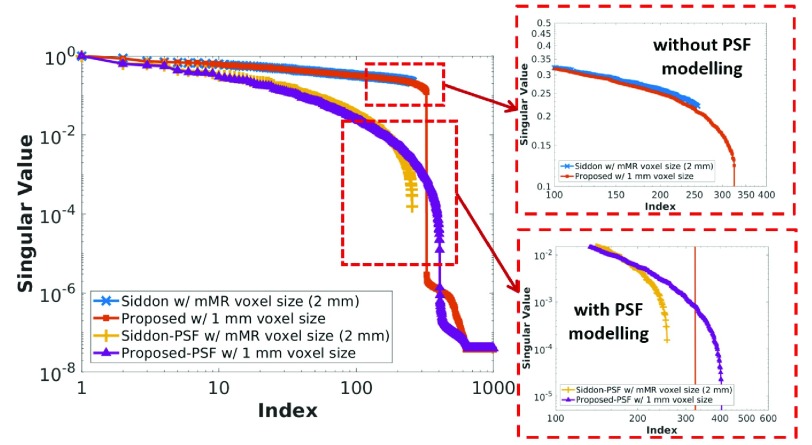
Singular values of the standard (2 mm voxel size) and the proposed (1 mm voxel size) system matrices, without and with PSF modeling, for a patch in the center of the FOV.

It could be argued that because of the simplicity of the method, the benefits of using smaller voxel sizes could be negligible. With the aim of showing that the modified system matrix can recover higher spatial frequencies than the standard system matrix, provided that they are available in the data, we computed the singular values of the standard and modified system matrix for a $16\times 16\times 8$ mm^3^ patch in the center of the field of view (FOV). In Fig. 2 the singular values of the standard (2 mm voxel size) and the proposed (1 mm voxel size) system matrices, without and with PSF modeling, are shown. It can be seen that the proposed system matrix is able to recover higher spatial frequencies than the standard method. This is more notable for the case of PSF modeling, where the inherent recovery of higher frequencies is enhanced.

Finally, the modified system matrix for smaller voxel sizes was validated using a 2 h scan of the NEMA IQ phantom acquired with a Biograph mMR scanner. A long scan was used in order to obtain reconstructed images with low noise where artifacts are easily visible. The images were reconstructed using the standard mMR PET voxel size of $2.09\times 2.09\times 2.03$ mm^3^ and a $1.05\times 1.05\times 1.00$ mm^3^ voxel size for the higher resolution image. Fig. 1 shows how the proposed method (right image) can overcome the artifacts problem (middle image) of reconstructing the image in a higher resolution matrix with a Siddon projector.

## III. MR-Guided Penalized Likelihood Reconstruction

PL reconstruction, also known as MAP, has been developed to reduce noise using prior assumptions regarding the unknown image. In order to achieve this, the image is reconstructed by maximizing a Poisson log-likelihood function with a penalty term

\begin{equation*} \hat {\theta } = \mathop {\mathrm {argmax}} _\theta \left \{{ L(m\vert \theta)-\beta U(\theta) }\right \}\tag{3}\end{equation*}

where $\theta $ is the unknown image, $m$ is the measured data, and $L(m\vert \theta)$ is the log-likelihood function for Poisson data. The energy function $U(\theta)$ is designed to penalize large differences between the estimated voxel values and their neighbors, since the image is expected to be smooth with the exception of boundaries. The hyperparameter $\beta $ controls the level of regularization or smoothness in the reconstruction.

The energy function $U(\theta)$ depends on the local differences between every voxel $j$ and the neighbors $\mathcal {N}_{j}$ of each such voxel

\begin{equation*} U(\theta) = \sum _{j}^{N}{\sum _{k \in \mathcal {N}_{j}}{\xi _{jk} w_{jk} \psi (t)}}\tag{4}\end{equation*}

where $N$ is the number of voxels in the reconstructed image, $k$ is a voxel in the set of neighbors $\mathcal {N}_{j}$ of pixel $j$, $\psi $ is the potential function, $t$ is a measure of the difference of intensities between voxel $j$ and $k$, and $\xi _{jk}$ and $w_{jk}$ are weights for the local difference based on the proximity and similarity of voxels $j$ and $k$.

### A. Lange Prior

A common penalty function is the quadratic or Tikhonov prior, where the potential function used is the quadratic function $\psi (t)=(1/2)t^{2}$. This prior generates an over-smooth image that blurs real edges of the image and, for that reason, edge-preserving priors have been proposed by many authors, such as total-variation (TV) [29]–[31]. A flexible edge-preserving function, that unlike TV has a continuous second-derivative, is the Lange or Fair function [30]

\begin{equation*} \psi (t) = \delta \left [{\frac {\left |{t}\right |}{\delta } - \log \left ({1+\frac {\left |{t}\right |}{\delta }}\right)}\right]\tag{5}\end{equation*}

where $\delta $ is a hyperparameter that controls the level of edge-preservation in the prior. With a large $\delta $ value the potential function behaves similarly to the quadratic, while for a small value it behaves similarly to TV.

Commonly, the difference $t$ between a voxel and its neighbors is the local intensity difference $\theta _{j}-\theta _{k}$. However, this approach is not robust to avoid penalizing real edges in the image and is very sensitive to the $\delta $ hyperparameter. To overcome this issue, the use of patch-based penalty functions was proposed by Wang and Qi [32], where to compute the difference between pixels $j$ and $k$, the intensity difference between the voxels of a square box (patch) centered in pixel $j$ and the respective voxels of a patch centered in $k$ is computed.

**Fig. 3.**
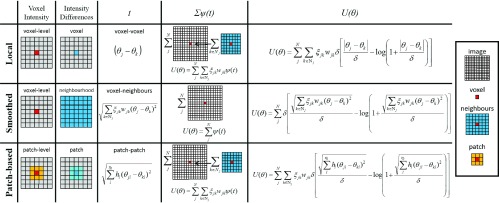
Description of three different possibilities to implement a Lange prior: standard or local, smoothed, and patch-based. For each implementation, the value used for the voxel $j$, the voxel $k$, and the intensity difference between them is shown; as well as the level (voxel, patch, neighbors, and image) at which the intensity differences are sum in the potential function $\psi $ and in the energy function $U$.

Inspired by the smoothed total variation [31] that it has been widely evaluated as a prior in the context of PET image reconstruction [24], a smoothed Lange prior is proposed in this paper. In this case, the potential function is evaluated with the root mean square difference between a voxel $j$ and all its neighbors, in contrast to the standard version where the potential function is evaluated for each local difference between voxel $j$ and each neighbor pixel $i$. This makes the energy function more robust to distinguish between noise and edges, and less sensitive to the $\delta $ hyperparameter, while being less computationally intensive than the patch-based version. Furthermore, for the smoothed Lange prior, $|t|$ is less likely to be 0 (although still possible) as it is computed over a set of neighbors instead of being local differences between voxels (see Fig. 3), and this therefore avoids possible problems when $\delta $ is also close to 0. An empirical comparison between the Lange and smoothed Lange priors can be found in the supplementary material.

The energy function for the smoothed Lange prior is then defined by

\begin{equation*} U(\theta) = \sum _{j}^{N}{\psi \left ({\sqrt {\sum _{k \in \mathcal {N}_{j}}{\xi _{jk} w_{jk} \left ({\theta _{j}-\theta _{k}}\right)^{2}}}}\right)}\tag{6}\end{equation*}

where $\psi $ is the Lange potential function of 5. In Fig. 3, the difference between the standard (local), the smoothed and the patched-version of the Lange prior [33] are summarized.

For the optimization of (3) we employed Green’s one step late (OSL) MAP-EM algorithm [34], where the ML-EM algorithm is modified by including an additive term in the sensitive image, which consists of the first derivative of the penalty function evaluated at the previous iteration. The derivative of the smoothed Lange prior is then needed

\begin{equation*} \frac {\partial U(\theta)}{\partial \theta _{j}} ={\frac {\sum _{k \in \mathcal {N}_{j}}{\xi _{jk} w_{jk} \left ({\theta _{j}-\theta _{k}}\right)}}{\delta +\sqrt {\sum _{k \in \mathcal {N}_{j}}{\xi _{jk} w_{jk} \left ({\theta _{j}-\theta _{k}}\right)^{2}}}}}.\tag{7}\end{equation*}

The OSL approach was employed because it has been widely used and its implementation is straightforward. However, the OSL MAP-EM algorithm has been shown to converge to the MAP solution only for potential functions that have a bounded derivative and provided that the regularization hyperparameter $\beta $ is small enough to avoid negative values in the denominator of the MAP-EM algorithm [30]. For this reason, special attention needs to be paid to the hyperparameter selection to avoid failure of convergence and negative values in the reconstructed images. In this paper, we have only used $\beta $ values that met this requirement. An alternative approach that guarantees convergence was proposed by De Pierro [35], although its convergence is slower.

### B. MR-Guided MAP

Standard PET MAP reconstruction has the problem of smoothing real edges in the image and, as a consequence, reducing the contrast of the reconstructed images, even for edge-preserving priors. In order to avoid the loss of boundary information, the use of anatomical information provided by other imaging technologies, such as MRI, has been proposed and evaluated widely [18], [24]. Different approaches and priors have been previously proposed to incorporate MR anatomical information in MAP reconstructions.

A well-established anatomical prior is the Bowsher prior, which selects (using a binary similarity weight) only a set of neighboring voxels to be included in the penalty function based on the anatomical information available [15]. A modified Bowsher method (asymmetric) was proposed in [17], which has shown a superior quantitative accuracy than the standard reconstruction methods [18]. A known disadvantage of the Bowsher method is that it is vulnerable to mismatches between the activity distribution and the anatomical structures. In [24], we evaluated more sophisticated priors that address the limitations of the Bowsher prior in the presence of PET-MR mismatches. However, the Bowsher method achieved the best performance in terms of PVC, showing lower normalized root mean square error (NRMSE) in the gray and white matter for simulated data.

Since this paper focuses on the enhancement of PVC, here we use the modified (asymmetric) Bowsher method [17], which have the additional advantage of being simple to incorporate different priors, such as the smoothed Lange prior.

In our implementation of the smoothed Lange penalty function, the similarity weight $w_{jk}$ of (6) takes a binary value, thereby enabling smoothing within anatomical regions and avoiding smoothing across anatomical boundaries. To compute the weights $w_{jk}$, the $B$ most similar neighbors to voxel $j$ in the anatomical image (i.e., MRI) are set with a value of 1, while the others with 0. For the weight $\xi _{jk}$, we used the inverse of the Euclidean distance between voxel $j$ and $k$. These proximity weights were normalized so that the sum of the proximity weights $\xi _{jk}$ for the set of neighbors $\mathcal {N}_{j}$ is 1. This way, the same regularization hyperparameter $\beta $ can be used for different neighborhood sizes. When the voxel sizes are different, the size of the patch in mm must be the same in order to use the same $\beta $ value.

## IV. Evaluation

Simulated and real patient data were used for the assessment of the PVC performance of each method.

### A. Simulation Study

Ten realizations of a brain scan were calculated using a brain phantom based on the Brainweb phantom [36], which was used to create an [^18^F]FDG PET phantom, an attenuation map, and a T1-weighted image. For the PET phantom, we used the discrete anatomical model of a normal brain available in the Brainweb dataset, which is a volume where each voxel is labeled with one tissue type out of nine classes available. Uptake values for each of the tissue types were set to match the contrast of a typical [^18^F]FDG PET scan, with uptake in the gray matter being four times greater than the uptake in the white matter. The attenuation map was defined using only two tissue types: 1) soft tissue and 2) bone. For the T1-weighted simulation, the Brainweb tool was used [36]. The voxel size of the phantom was $1\times 1\times 1$ mm^3^.

The PET scan was simulated by smoothing the [^18^F]FDG phantom with a 4.3 mm FWHM kernel (corresponding to the spatial resolution of the mMR scanner) [37] and projecting the image into a span 11 sinogram using the mMR scanner geometry. Then the resulting sinograms were multiplied by the attenuation factors, obtained from the attenuation map, and by the normalization factors of the mMR scanner [38]. Next, Poisson noise was introduced by simulating a random process for every sinogram bin, obtaining the sinogram with true events. A uniform sinogram multiplied by the normalization factors was used for the randoms and a smoothed version of the emission sinogram for the scatters. Finally, Poisson noise was introduced to randoms and scatters and added to the trues sinogram.

This process was performed for each of the ten realizations. All of them were simulated with a total of $4.7\times 10^{8}$ prompt counts, including 25% random events and a scatter fraction of 28%, in order to match the statistics of the real patient data set described in the following section.

### B. Real Patient Data

**Fig. 4.**
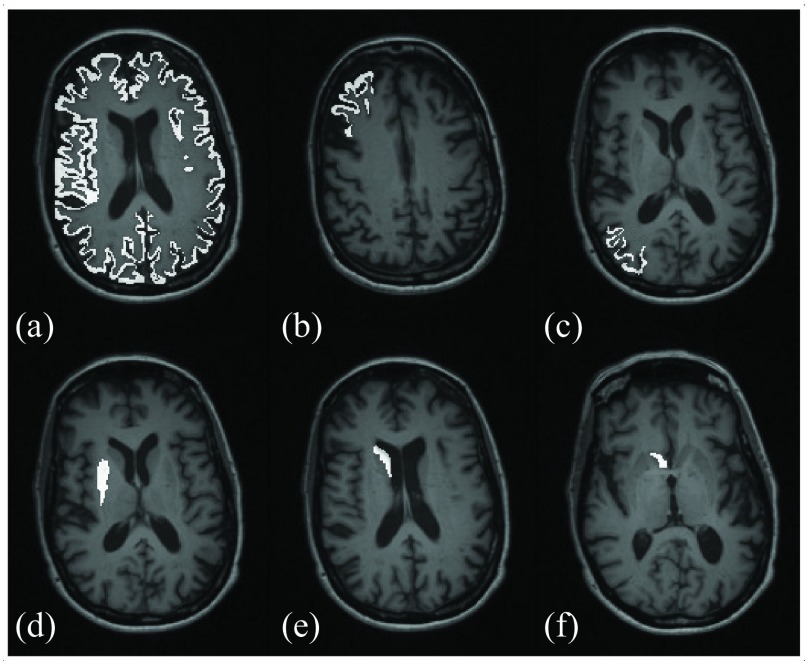
T1-MPRAGE image of the real patient data overlaid with the ROIs used to assess the image quality and PVC: (a) cortical gray matter, (b) middle frontal gyrus, (c) inferior parietal lobule, (d) putamen, (e) caudate, and the (f) nucleus accumbens.

Real patient data acquired with the mMR scanner for an [^18^F]FDG brain study was used to evaluate the enhancement of PVC in the cortical and subcortical gray matter. The data were from an AD patient injected 80 min before the scan with 228 MBq. The scan duration was 23 min and a total of $4.7\times 10^{8}$ prompt counts were acquired. T1-MPRAGE data was acquired simultaneously with a voxel size of $1.05\times 1.05\times 1.1$ mm^3^, which was registered to an MLEM reconstructed image using FSL-FLIRT (FMRIB’s Linear Image Registration Tool) [39], [40] in order to avoid any misalignment between the PET and the MRI images. The contrast and coefficient of variation in the gray matter for a set of cortical and subcortical regions, which can be seen in Fig. 4, were computed to compare the different methods. For the cortical gray matter, the middle frontal gyrus and the inferior parietal lobule were used, the latter being one of the affected regions in the early stages of AD as observed in [^18^F]FDG studies [41]; while for the subcortical regions, the caudate, the nucleus accumbens, and the putamen were used. All the ROIs were segmented with freesurfer [42].

### C. Image Reconstruction

Simulation and real data studies were reconstructed with our own software [43]. MLEM, MAP, and MR-guided MAP reconstructions of each data set were carried out with 400 iterations for the simulated data and 400 iterations for the real patient data, for the standard mMR PET voxel size and for the MRI voxel size, which we call MRvox from here on, using the modified system matrix of Section II. Both MAP and MR-guided MAP reconstructions used the smoothed Lange prior defined in (6). In the MR-guided MAP reconstructions, the similarity weights were obtained with the Bowsher method and were computed from the T1-weighted image using 40 most similar neighbors in a $5\times 5\times 5$ neighborhood for the two different voxel sizes. For the standard PET voxel size, the T1-weighted image was downsampled to match the PET matrix.

The reconstructions were performed without and with resolution modeling implemented in image space [11]. The reconstructions without resolution modeling consisted of a narrow Gaussian PSF of 2.5 mm FWHM and a Siddon projector, while for the resolution modeling the PSF was of 4.5 mm FWHM. These parameters were chosen to match the reconstructions without and with resolution modeling of the Siemens e7 tools [44]. For the real data, the correction sinograms were computed with e7 tools.

Finally, the data sets were also reconstructed similarly to the method routinely used clinically, but without subsets. The MLEM reconstructions were performed with 60 iterations, while the MLEM reconstructions with resolution modeling were run for 80 iterations, broadly equivalent to 3 and 4 iterations with 21 subsets, respectively (clinical set up). In both cases, the images were smoothed with a 4 mm FWHM Gaussian filter.

### D. Parameter Selection

In the MAP reconstructions with the smoothed Lange prior there are two hyperparameters to select, the standard MAP regularization parameter $\beta $ and the parameter $\delta $ of the Lange potential function that controls edge preservation. A set of both parameters were selected empirically for the different reconstructions but taking into account a set of considerations to do the selection in a reproducible fashion. It can be observed in (5) that the $\delta $ hyperparameter affects the scaling of the function and therefore the $\beta $ value needs to be modified to achieve a similar amount of regularization. For this reason we implemented a hyperparameter selection method, where the $\beta $ values are scaled automatically based on the $\delta $ value.

First, a $\delta $ value and a range of $\beta $ parameters, which generates different levels of regularization, were selected and named $\delta _{0}$ and $\beta _{0}$, respectively. The $\beta _{0}$ values were selected to be 0.1, 0.2, 0.5, 1, and 2 times the mean value of the sensitivity image in the center of the FOV. Therefore, these hyperparameters are also independent of the system matrix used (standard or modified for the MRvox voxel size) because in OSL MAP-EM the penalty term is additive to the sensitive image.

**Fig. 5.**
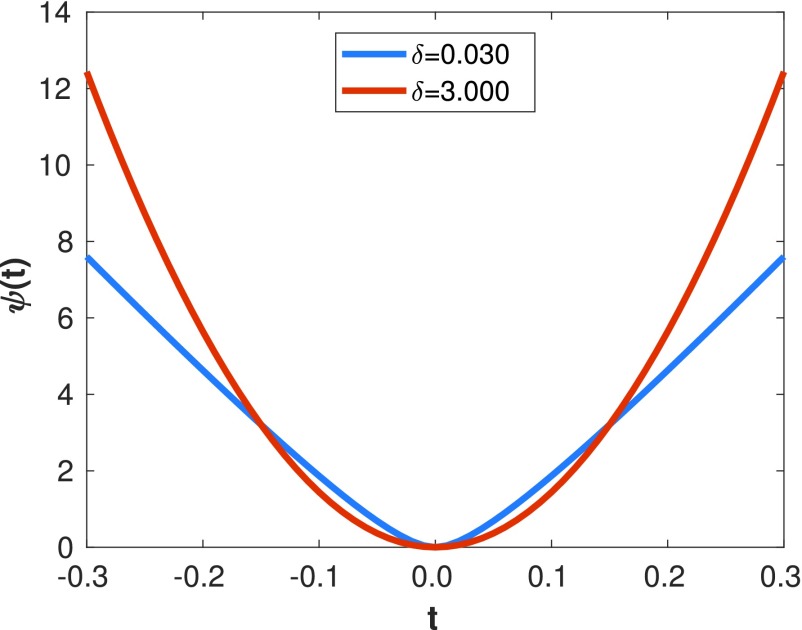
Lange potential function for two different $\delta $ values and scaled using $k(\delta)$ for a $\Delta _{\theta }$ of 0.3.

Second, a $\delta _{0}$ value was selected empirically to achieve a TV behavior in the prior, as can be seen in the shape of the potential function in blue in Fig. 5, and to be effective with the $\beta _{0}$ values selected previously. This parameter is related with the range of intensity differences in the image that will be penalized ($\Delta _{\theta }$), which we set at 25% the dynamic range of the image. $\delta _{0}$ then was set at $0.1\times \Delta _{\theta }$.

Finally, greater values of $\delta $ were chosen to obtain a quadratic like prior and the $\beta $ values were scaled to obtain a similar amount of regularization to that obtained with $\delta _{0}$

\begin{equation*} \beta = k(\delta) \beta _{0} \quad ~ k(\delta) = \frac {\Delta _{\theta }+\delta _{0}}{\Delta _{\theta }+\delta }\tag{8}\end{equation*}

where $\beta $ is the final regularization hyperparameter used in the regularization term and $k(\delta)$ is the scaling parameter that depends on the $\delta $ value. In Fig. 5, a plot of the Lange potential function for two different $\delta $ values and scaled using $k(\delta)$ is shown for a $\Delta _{\theta }$ of 0.3.

The Bowsher parameters were fixed as described in the previous section.

### E. Metrics

For the real and simulated data sets, the contrast and coefficient of variation in the cortical gray matter, the middle frontal gyrus, the inferior parietal lobule, the caudate, the nucleus accumbens, and the putamen were computed. The ROIs were segmented with freesurfer (in Fig. 4 for the real patient data), but for the case of simulated data the regions were restricted to include only gray matter voxels (as in the original phantom) in order to avoid segmentation errors.

The contrast of the selected region against the white matter was used as the main metric to evaluate the PVC obtained with the different methods under evaluation

\begin{equation*} C_{RW}^{l}=\frac {\frac {1}{N_{R}}\sum _{j}^{N_{R}}{\theta _{j}^{l}}} {\frac {1}{N_{W}}\sum _{k}^{N_{W}}{\theta _{k}^{l}}}\tag{9}\end{equation*}

where $\theta _{j}^{l}$ is the value of voxel $j$ of the reconstructed image at iteration $l$, $j$ is one of the $N_{R}$ voxels of the ROI being analyzed and $i$ is one of the $N_{W}$ voxels that make up the ROI of the white matter.

The coefficient of variation in each of the six ROIs was employed as a noise metric

\begin{equation*} {\mathrm{ COV}}^{l}= \frac {1}{\frac {1}{N_{R}} \sum _{j}^{N_{R}} {\theta }_{j}^{l}} \sqrt {\frac {1}{N_{R}-1} \sum _{j}^{N_{R}}\left ({{\theta }_{j}^{l} - \frac {1}{N_{R}} \sum _{j}^{N_{R}} {\theta }_{j}^{l} }\right)^{2}}.\tag{10}\end{equation*}

All the metrics were computed in the MRvox voxel size. For the standard voxel size reconstructions, the images were interpolated into the higher resolution matrix before computing the metrics. For the simulated data set, these regional metrics were computed only for one realization.

In addition, an error-variance analysis was performed using ten noise realizations. The NRMSE at an ROI level was used as error metric, which can be considered as an alternative measure to bias, and the COV at voxel level as a metric of noise. Both metrics were computed for each ROI for every iteration. The NRMSE for an individual ROI is defined by

\begin{equation*} {\mathrm{ NRMSE}}^{l}_{R} = \sqrt {\frac {1}{M}\sum _{m=1}^{M}{\frac {\left ({\overline {\theta _{Rm}^{l}}-\overline {\theta _{R}^{\mathrm{ true}}} }\right)^{2}}{\overline {\theta _{R}^{\mathrm{ true}}}}}}\tag{11}\end{equation*}

where $\overline {\theta _{Rm}^{l}}$ is the mean voxel value in the ROI $R$ of the reconstructed image at iteration $l$ for the noise realization $m$, $M$ the total number of realizations, and $\overline {\theta _{R}^{\mathrm{ true}}}$ is the mean voxel value in the ROI $R$ for the phantom.

The mean ensemble COV of every voxel in a given region (${\mathrm{ COV}}_{ER}$) was used to measure noise

\begin{equation*} {\mathrm{ COV}}^{l}_{ER}= \frac {1}{N_{R}} \sum _{j}^{N_{R}} {\frac {\sqrt {\frac {1}{M-1}\sum _{m=1}^{M}{\left ({{\theta }_{jm}^{l}-\overline {\theta _{j}^{l}}}\right)^{2}}}} {\overline {\theta _{j}^{l}}}}\tag{12}\end{equation*}

where ${\theta }_{jm}^{l}$ is the value of voxel $j$ at iteration $l$ for the noise realization $m$, the voxel $j$ is one of the $N_{R}$ voxels of the ROI under analysis, and $\overline {\theta _{j}^{l}}$ is the ensemble mean value of voxel $j$.

## V. Results

### A. Simulation Study

**Fig. 6.**
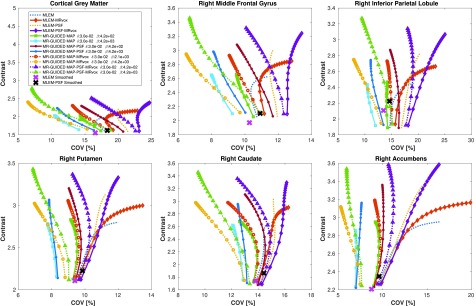
Contrast versus COV in six ROIs as a function of the iteration number for MLEM, MR-guided MAP, and MR-guided MAP with PSF modeling reconstructions using standard and MR voxel sizes, for a single noise realization of the simulation study. The metrics were sampled every 20 iterations, starting at iteration 20 and finishing at iteration 400. The contrast in the ground truth was 4.

**Fig. 7.**
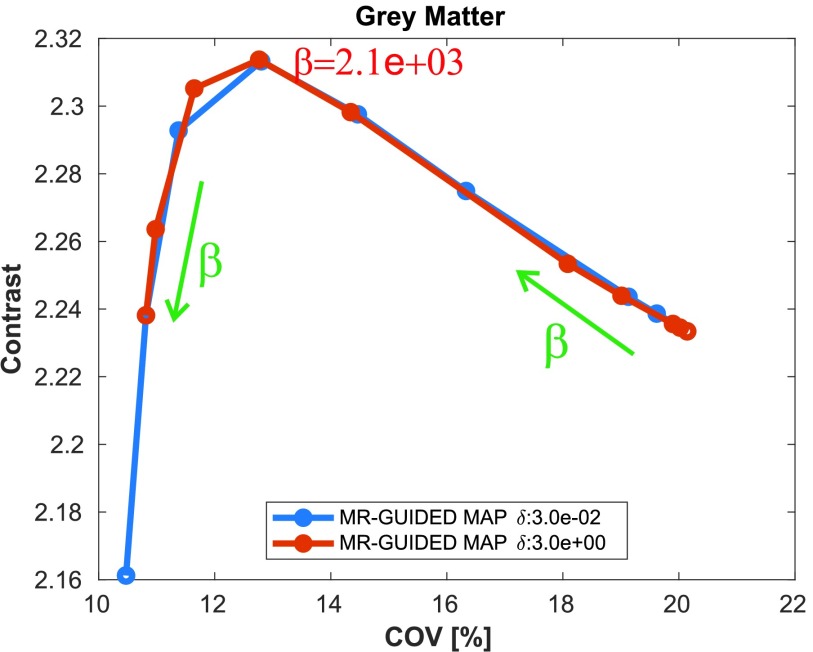
Contrast versus COV in the cortical gray matter as a function of the regularization hyperparameter $\beta $ for two different $\delta $ values for MR-guided MAP reconstruction with PSF modeling and standard voxel sizes. All the images were evaluated at iteration number 400. The $\beta $ value grows in the direction of the green row and the $\beta $ value for maximum contrast is in red text. The true contrast in the phantom was 4.

In Fig. 6, the contrast and regional COV values are shown as a function of the iteration number for a single realization for each of the reconstruction methods evaluated: MLEM and MR-guided MAP without and with resolution modeling for standard and MRvox voxel sizes. Each of the different MR-guided MAP reconstructions are shown for only two different selections of regularization hyperparameters, with a fixed $\delta $ value and two different $\beta $ values. The parameter selection was performed so that a good performance in terms of contrast (therefore good PVC) is obtained, as well as good image quality by visual inspection. An exploration of the regularization hyperparameters for the MR-guided MAP reconstruction with resolution modeling and standard voxel sizes is shown in Fig. 7, where it can be seen that for MR-guided reconstructions the $\delta $ value chosen did not have any notable impact on the performance since it was possible to find a $\beta $ value for each of the $\delta $ evaluated ($\delta =0.03$ for a pseudo TV prior and $\delta =3$ for a pseudo quadratic prior) so as to obtain matched performance. For this reason in Fig. 6, we only used $\delta =0.03$. The $\beta $ value for maximum contrast (in red text in Fig. 7) was one of the values chosen for the comparison.

**Fig. 8.**
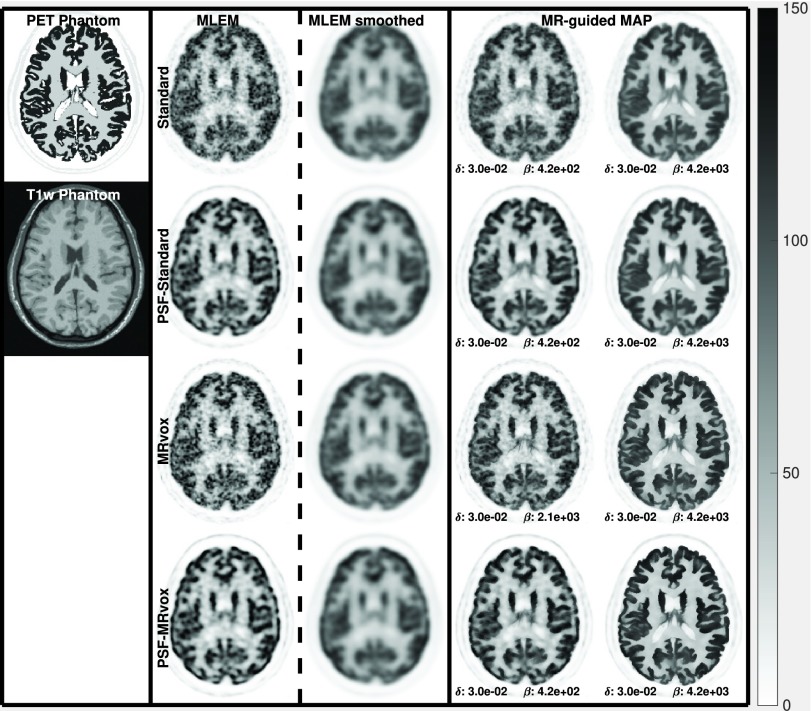
MLEM, MR-guided MAP, and MR-guided MAP with PSF modeling reconstructed images for standard and MRvox voxel sizes at iteration number 400, for the simulation study. MLEM reconstructed images as performed in clinical routine are also presented in the MLEM smoothed column. The MR-guided MAP reconstructions in the bottom row (*PSF-*MRvox) show the best contrast and resolution. The color scale is in arbitrary units.

In Fig. 8, the reconstructed images are shown for the same reconstructions as in Fig. 6. In the first column, the phantom is shown. The MLEM images at iteration 400 are displayed in the second column, while in the third column the MLEM reconstructions as used clinically are shown (60 iterations without PSF and 80 iterations with PSF modeling, and a 4 mm FWHM Gaussian filter). The MR-guided MAP reconstructions are shown in the fourth and fifth columns. For every type of reconstruction, the images are shown from top to bottom for the standard voxel size, the PET standard voxel size and resolution modeling, MRvox voxel size and MRvox voxel size and resolution modeling.

In Figs. 6 and 8, it can be observed that the MR-guided reconstructions successfully reduced the noise, as was expected and shown in previous work, especially for the case of simulated data where the brain structures match perfectly [24]. Importantly, the use of MRvox voxel size enhanced the contrast of the images for all the reconstruction methods. However, for the MLEM reconstructions it came at the cost of an increase of noise. The MR-guided reconstructions with MRvox voxel size not only enhanced the contrast but also the noise was reduced compared to the standard voxel size. A possible reason for the latter is that the spill over of counts outside the gray matter structures is reduced do to a more accurate location of the edges and to the resolution recovery obtained with resolution modeling.

The reconstructions with resolution modeling for standard and MRvox voxel sizes obtained a higher contrast but at the cost of higher COV in the striatum due to Gibbs artifacts, which are common overshoot artifacts observed in small objects when using resolution modeling [14]. The use of MR-guidance avoided these artifacts.

**Fig. 9.**
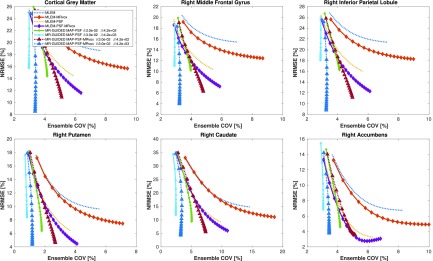
NRMSE versus ensemble COV over ten noise realizations for MLEM, MR-guided MAP, and MR-guided MAP with PSF modeling reconstructions for standard and MRvox voxel sizes as a function of the iteration number.

**Fig. 10.**
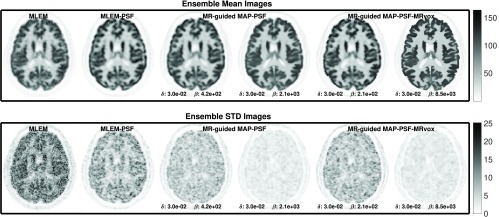
Mean and standard deviation images over ten noise realizations for MLEM, MLEM-PSF, MR-guided MAP-PSF ($\beta = {4.2\times 10^{2}}$ and $\beta = {2.1\times 10^{3}}$), and MR-guided MAP-PSF-MRvox ($\beta = {2.1\times 10^{2}}$ and $\beta = {8.5\times 10^{3}}$) at iteration number 400. The color scale is in arbitrary units.

For the error-variance analysis performed with multiple realizations, the NRMSE versus ensemble COV in the six chosen ROIs are presented in Fig. 9 as a function of the iteration number, for the same reconstructions hyperparameters as the ones for a single realization. Here, also the MR-guided reconstructions using the MRI image in its original resolution outperformed the reconstructions with the standard voxel size. The reconstructions with MRvox and PSF obtained the lowest error and the second lowest COV for the same regularization hyperparameters, showing that in combination they form a powerful PVC method. In Fig. 10, the mean images of the ten noise realizations are presented in the top row for the MLEM, MLEM-PSF, MR-guided MAP-PSF, and MR-guided MAP-PSF-MRvox. The two latter are shown for the stronger regularization parameter of the two shown in Fig. 9. In the bottom row, the standard deviation at voxel level is also shown, where it can be seen that the MR-guided MAP-PSF obtained lower noise when using standard voxel size compared to the MRvox voxel sizes.

**Fig. 11.**
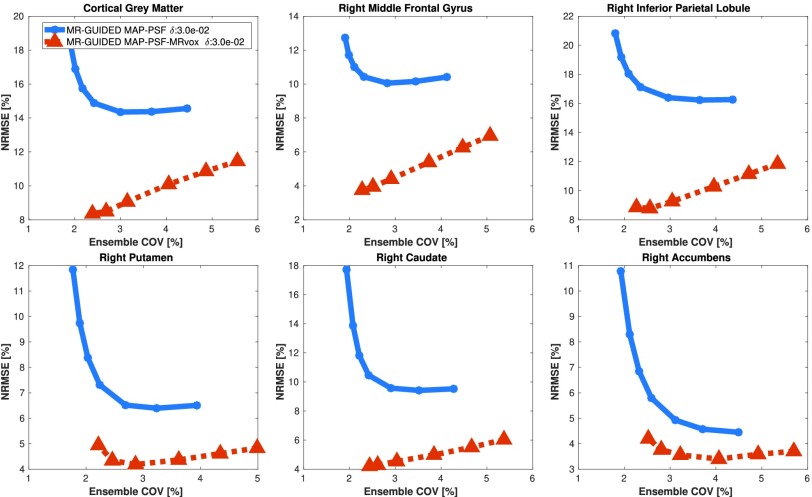
NRMSE versus ensemble COV over ten noise realizations for MR-guided MAP with PSF modeling reconstructions for standard and MRvox voxel sizes at iteration number 400 as a function of the regularization parameter $\beta $. The $\beta $ values used were ${2.1\times 10^{2}}$ (only for the standard voxel size), ${4.2\times 10^{2}}$, ${8.5\times 10^{2}}$, ${2.1\times 10^{3}}$, ${4.2\times 10^{3}}$, ${8.5\times 10^{3}}$, and ${4.2\times 10^{4}}$, from left to right. A fixed $\delta $ value of $3\times 10^{-2}$ was used.

Finally, in order to draw a comparison between the two best performing methods (MR-guided MAP-PSF and MR-guided MAP-PSF-MRvox), they were evaluated for a wider range of $\beta $ values (from $\beta = {2.1\times 10^{2}}$ to $\beta = {4.2\times 10^{4}}$). In Fig. 11, the NRMSE versus ensemble COV in the six chosen ROIs are shown at iteration 400 as a function of the regularization hyperparameter $\beta $, for the two mentioned methods. This figure shows that the use of MRvox voxel sizes reduces considerably the error in MR-guided reconstruction for the same noise level.

### B. Real Patient Data

**Fig. 12.**
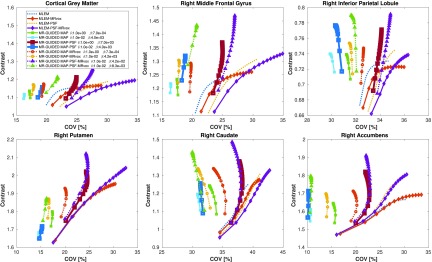
Contrast versus COV in six brain regions as a function of the iteration number for MLEM, MR-guided MAP, and MR-guided MAP with PSF modeling reconstructions using standard and MR voxel sizes for the real data study. The metrics were sampled every 20 iterations, starting at iteration 20 and finishing at iteration 400.

In Fig. 12, the contrast and COV metrics are shown for the same methods evaluated in the simulation study for standard and $1.05\times 1.05\times 1.0$ mm^3^ (MRvox) voxel sizes, where similar results to those obtained with simulated data are observed. The MR-guided MAP reconstructions reduced the noise levels considerably. Despite the good recovery of the cortical and subcortical brain structures, a loss of contrast is seen for the standard voxel size. The latter is avoided when reconstructing with MRvox voxel sizes, where better delineation of the structures of the brain and an enhancement of the contrast is achieved, obtaining similar contrast values as the standard MLEM reconstruction. The inclusion of resolution modeling proved to be essential in order to obtain PVC, but once again the use of MRvox voxel sizes showed an important additional enhancement of the contrast for the same noise level (between 15% and 20%). The noise reduction with MR-guided methods was less considerable than for the simulation study.

**Fig. 13.**
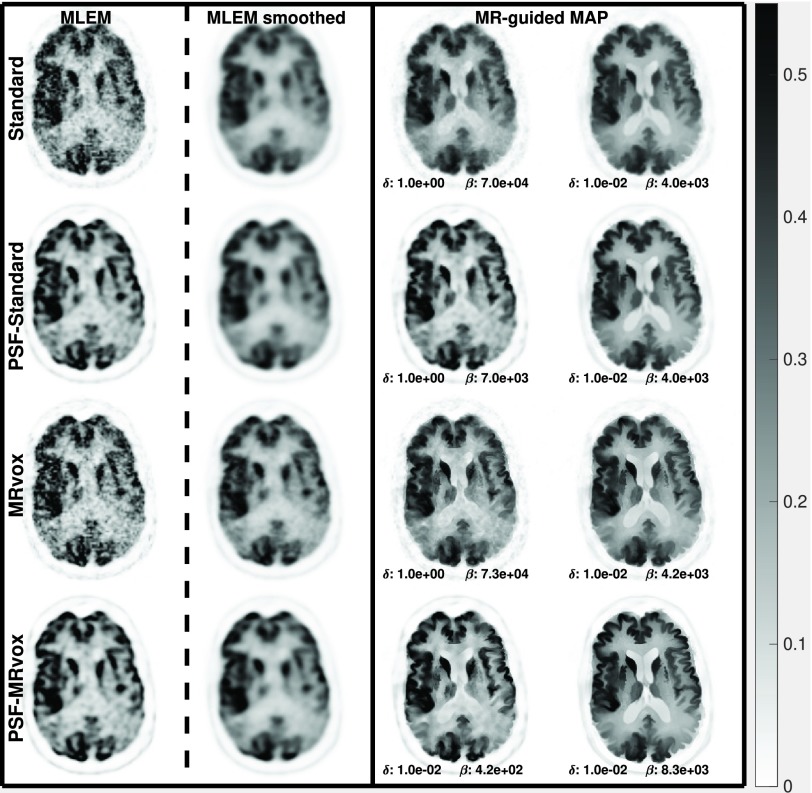
MLEM, MR-guided MAP, and MR-guided MAP with PSF modeling reconstructed images for standard and MR voxel sizes at iteration number 400 for the real data study. MLEM reconstructed images as performed in clinical routine are also presented in the MLEM smoothed column. The color scale is in arbitrary units.

In Fig. 13, the reconstructed images of the real data study are shown for the same reconstruction methods evaluated in Fig. 12. The use of MRvox voxel sizes achieved better contrast when comparing equivalent methods. For MR-guided reconstructions, the loss of contrast due to the regularization was successfully compensated for by using the MRI anatomical information in its original resolution. As was shown with simulated data, the combination of MR-guided MAP reconstruction with PSF modeling and MRI voxel sizes enhances considerably the contrast while reducing and controlling the noise level and artifacts seen in resolution modeling.

## VI. Discussion

**Fig. 14.**
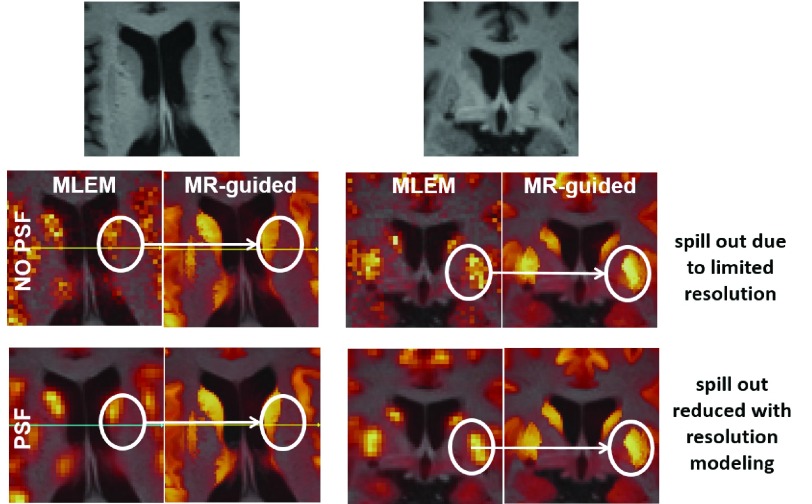
Impact of resolution modeling in MR-guided reconstructions. In the top row, a patch on a transverse (left) and coronal (right) slice of the T1-weighted image. In the middle row, the same slices for the MLEM and MR-guided reconstructions without resolution modeling. In the bottom row, images reconstructed with resolution modeling.

For both real and simulated data, an important enhancement of the contrast was observed when using the MRI anatomical information in its native resolution (MRvox voxel sizes) in MR-guided reconstructions. The modeling of the PSF was a very important factor for achieving good PVC and enhancement of the contrast for all the methods under evaluation, but the best performance in terms of PVC was obtained when resolution modeling was combined with MR-guided reconstructions with MRvox voxel sizes. This combination prevents the spill out of activity and, therefore, avoids the smoothing outside the correct anatomical boundaries. Moreover, the use of MR-guided reconstructions eliminates the Gibbs artifacts that are introduced by resolution modeling methods [12], [13]. Fig. 14 looks into this effect, where two transverse and coronal patches of the reconstructed images are fused with the T1-weighted image. For the case of reconstructions without resolution modeling (NO PSF row in the figure), a non-negligible amount of activity is located outside the brain structures where the activity was presumably located (based on the PSF reconstructions of the bottom row). This is a consequence of the low resolution of the PET images and it is exacerbated by the smoothing applied in the regularization, even when anatomical boundaries are used as prior information, and for that reason an important loss of contrast is observed in those cases where resolution modeling is not implemented.

When using multiple realizations to compare MR-guided MAP-PSF reconstructions with standard and MRvox voxel sizes, a similar enhancement of the contrast to that obtained for a single realization was observed. However, a lower ensemble COV was obtained for the standard voxel size and this is also seen in the standard deviation images of Fig. 10. This could be due to slightly different regularization strength. In the proposed implementation and hyperparameter selection the same regularization hyperparameters produced a similar, although not the same, regularization strength for different methods and different voxel sizes, as can be observed in the reconstructed images such as in Fig. 8. Fig. 11 confirms the better performance of using MRvox voxel sizes for matched regularization levels by comparing these two methods for a larger range of hyperparameters, showing that for the same level of COV a considerably lower error is achieved.

For real data, it cannot be assured that the improvement of the contrast is in fact improving the quantification in the images by accurately correcting the PVE. However, when the image is studied locally as in the patches shown in Fig. 14, there is a very high correlation between the gray matter in the T1-weighted image and the [^18^F]FDG uptake in the PET image. This is even more noticeable when resolution modeling is used. In addition, when we compare the results between simulated and real data, a good enhancement of the contrast was obtained in both cases, although it was more modest for real data. The use of MR-guided MAP-PSF with MRvox voxel sizes produced a further reduction of the noise for simulated data, but this was not observed for real data. A possible reason for these results is the perfect boundary matching between the PET and T1-weighted image phantoms in the simulated data.

Finally, the proposed method that allows the reconstruction of images with smaller voxel sizes is only necessary when ray-tracing approaches are used as projectors, especially for the Siddon algorithm used in this paper. Other ray-tracing algorithms with an embedded interpolation, such as the Joseph method [45], would still need a similar approach when the high resolution voxel sizes are approximately half the size of the standard voxel sizes or smaller. The use of PSF modeling can also make the downsampling matrix redundant, but then only reconstructions with resolution modeling could be performed and with a higher computational cost due to projecting high resolution images. For voxel-driven [46] or distance-driven [47] projectors, the modified system matrix would not be necessary but they have an even more considerable computational cost that scales with the reduction of the voxel size.

## VII. Conclusion

In order to be able to reconstruct the images with smaller voxel sizes than the standard for a given scanner, we proposed a simple and effective modification to a system matrix based on a ray-tracing projector, where a downsampling matrix is applied before the projector. Then, this system matrix was used in MR-guided MAP reconstructions with a modified Lange prior that included anatomical information with the Bowsher method. These reconstruction achieves a good enhancement of the contrast for both simulated and real data. The use of MRI voxel sizes combined with resolution modeling reconstructions proved to enhance PVC substantially, increasing 15%–20% the contrast in the striatum, without introducing artifacts and reducing the noise in the images.

To conclude, we showed the importance of the use of MRI voxel sizes when reconstructing PET images with MR-guided MAP methods since it shows an enhancement of the contrast and a reduction of the errors due to PVE as it was shown for simulated data. These improvements are due to a better use of the anatomical information by using it in its native resolution, which allows the preservation of high frequency detail; and because of the use of a higher resolution matrix, where higher frequencies can be recovered, especially for reconstructions with resolution modeling.
